# Seizing the Connection: Exploring the Interplay Between Epilepsy and Glycemic Control in Diabetes Management

**DOI:** 10.7759/cureus.45606

**Published:** 2023-09-20

**Authors:** Muhammad Daniyal Nadeem, Siraj Memon, Kashifa Qureshi, Umer Farooq, Unaib Ahmed Memon, FNU Aparna, Meet Popatbhai Kachhadia, FNU Shahzeen, Sameer Ali, Giustino Varrassi, Lakshya Kumar, Sumeet Kumar, Satesh Kumar, Mahima Khatri

**Affiliations:** 1 Medicine, Khyber Teaching Hospital, Peshawar, PAK; 2 Medicine, Liaquat University of Medical & Health Sciences, Jamshoro, PAK; 3 Medicine, CMH Lahore Medical College and Institute of Dentistry, Lahore, PAK; 4 Neurology and Internal Medicine, Liaquat University of Medical and Health Sciences, Jamshoro, PAK; 5 Medicine, Ghulam Muhammad Mahar Medical College, Sukkur, PAK; 6 Internal Medicine, Pandit Deendayal Upadhyay Medical College, Rajkot, IND; 7 Internal Medicine, Jinnah Sindh Medical University, Karachi, PAK; 8 Internal Medicine, Liaquat University of Medical and Health Sciences, Jamshoro, PAK; 9 Pain Medicine, Paolo Procacci Foundation, Rome, ITA; 10 General Medicine, Pandit Deendayal Upadhyay Medical College, Rajkot, IND; 11 Internal Medicine, Dow University of Health Sciences, Karachi, PAK; 12 Medicine and Surgery, Shaheed Mohtarma Benazir Bhutto Medical College, Karachi, PAK; 13 Medicine and Surgery, Dow University of Health Sciences, Karachi, PAK

**Keywords:** diabetes, seizures, bidirectional relationship, interplay, diabetes management, glycemic control, epilepsy

## Abstract

Epilepsy, a neurological disorder characterized by recurrent seizures, and diabetes, a metabolic disorder characterized by impaired regulation of glucose levels, are two distinct conditions that may appear unrelated at first glance. Nevertheless, recent scholarly investigations have revealed these entities' intricate and ever-evolving interplay. This review initially delves into the intricate interplay between epilepsy and its potential ramifications on glycemic control. Seizures, particularly those accompanied by convulsive manifestations, have the potential to induce acute perturbations in blood glucose levels via diverse mechanisms, encompassing the liberation of stress hormones, the emergence of insulin resistance, and the dysregulation of the autonomic nervous system. Comprehending these intricate mechanisms is paramount in customizing productive strategies for managing diabetes in individuals with epilepsy. On the contrary, it is worth noting that diabetes can substantially impact the trajectory and control of epilepsy. The correlation between hyperglycemia and an elevated susceptibility to seizures, as well as the potential for exacerbating the intensity of epilepsy, has been established. This narrative review offers a concise exposition of the intricate interplay between epilepsy and glycemic control within diabetes management. The objective of exploring reciprocal influences, underlying mechanisms, and common risk factors is to augment the clinical comprehension of this intricate interconnection. In essence, this acquired knowledge possesses the potential to serve as a guiding compass for healthcare professionals, enabling them to craft bespoke therapeutic approaches that enhance the holistic welfare of individuals grappling with the coexistence of epilepsy and diabetes.

## Introduction and background

Epilepsy and diabetes mellitus are two discrete and widely prevalent medical conditions that each bear substantial health burdens in their own right. Epilepsy, a neurological condition, manifests as a chronic disorder marked by repetitive, unprovoked seizures arising from atypical electrical discharges within the cerebral cortex. It exerts its influence upon an estimated populace of approximately 50 million individuals on a global scale, thereby establishing itself as one of the prevailing neurological disorders with widespread prevalence across the globe [1]. On the contrary, diabetes mellitus is a metabolic anomaly distinguished by compromised control of blood glucose levels, exerting its influence on an approximate population of 463 million individuals across the globe [2]. In light of recent scientific investigations, it has become evident that a nuanced and intricate relationship exists between epilepsy and diabetes despite their apparent dissimilarity. The intricate interconnectedness between these elements holds significant ramifications for the management of patients, thereby demanding a comprehensive comprehension of the intricate dynamics that govern their interplay.

The concurrent presence of epilepsy and diabetes presents noteworthy clinical, economic, and public health ramifications. As mentioned above, recognizing and comprehending the intricate interplay between these conditions is of utmost importance for many compelling reasons. Individuals suffering from the co-occurrence of epilepsy and diabetes experience a concomitant ailment burden that significantly influences their overall well-being. These individuals frequently encounter heightened healthcare utilization, escalated healthcare expenditures, and a reduced ability to carry out routine tasks [3,4]. Comprehending the intricate interplay between these conditions can facilitate healthcare providers in formulating more efficacious therapeutic approaches and augmenting the holistic welfare of these individuals. The coexistence of epilepsy and diabetes presents intricate difficulties in diagnosis and treatment. Seizure occurrences among individuals with diabetes may be inaccurately ascribed to hypoglycemia, resulting in the administration of unsuitable therapeutic interventions. On the contrary, it is imperative to acknowledge the potential oversight of the impact of hyperglycemia on seizures, as it may lead to inadequate provision of medical attention [5]. Comprehending the interconnection between these circumstances is imperative for precise diagnosis and productive administration. The interplay between treating a particular condition and its potential ramifications on another condition is significant. As an illustration, it is worth noting that the utilization of antiepileptic drugs (AEDs) and antidiabetic medications may engender an intricate interplay, thereby exerting an influence on the regulation of glycemic levels as well as the management of seizures [6,7]. Healthcare providers must exercise prudence and deliberation in their approach when formulating treatment plans for individuals who have both epilepsy and diabetes. Epilepsy and diabetes exhibit a convergence of risk factors, encompassing hereditary propensities, inflammatory mechanisms, and individual behavioral decisions [8]. Identifying and comprehending these mutually shared risk factors can offer profound insights into disease prevention and the implementation of timely intervention strategies that may prove advantageous for both afflictions.

The present narrative review aims to elucidate the complex and multifaceted relationship between epilepsy and glycemic control within diabetes management. The following principles guide the pursuit of its objectives: This review explores the intricate relationship between epilepsy and glycemic control, seeking to shed light on the reciprocal influence they exert upon one another. This necessitates a thorough investigation into the ramifications of seizures on blood glucose levels and the influence of hyperglycemia on the frequency and intensity of seizures. The narrative review aims to delve into the fundamental mechanisms that form the basis for the reciprocal interactions between epilepsy and diabetes. This necessitates an in-depth exploration of the intricate interplay between physiological, biochemical, and neuroendocrine pathways that establish the connections among these conditions. It is important to ascertain and engage in discourse regarding the common risk factors that give rise to the simultaneous presence of epilepsy and diabetes. A comprehensive examination of these shared characteristics can provide valuable insights for developing preventative measures and individualized treatment strategies. The comprehensive analysis will ultimately elucidate clinical ramifications stemming from the intricate relationship between epilepsy and diabetes. This encompasses the provision of guidance about the most advantageous treatment modalities, promoting interdisciplinary cooperation amongst healthcare practitioners, and accentuating the significance of patient enlightenment.

This narrative review endeavors to impart a holistic comprehension of the intricate correlation between epilepsy and glycemic regulation in diabetes management. By diligently attending to the objectives mentioned above, it strives to function as a highly esteemed repository for clinicians, researchers, and healthcare professionals, thereby making a substantial contribution to the enhancement of patient care and the attainment of superior outcomes for individuals contending with the dual challenges of epilepsy and diabetes.

## Review

Methodology

Literature Search and Selection Criteria

A thorough search of peer-reviewed literature was undertaken using relevant keywords, including "epilepsy," "diabetes," "glycemic control," "interplay," and related phrases, in electronic databases like PubMed, Scopus, and Google Scholar. 

Inclusion Criteria

Studies and papers examining the link between epilepsy and glycemic management in people with diabetes meet the inclusion criteria. Meta-analyses, reviews, and research articles shed light on the mechanisms and factors that operate in both directions. Studies explaining how AEDs and antidiabetic treatments affect managing epilepsy and controlling blood sugar. Articles examining the risk factors that diabetes and epilepsy share-publications focusing on interdisciplinary collaboration, clinical implications, and patient management.

Exclusion Criteria

Studies that address either diabetes or epilepsy without taking into account how the two conditions interact are excluded. Articles that cannot be read in English. Before the year 2000, written literature.

Data Extraction and Synthesis

From selected studies, pertinent data were culled, including details on standard risk factors, bidirectional influences, underlying processes, and clinical consequences. To give a thorough overview of the interactions between epilepsy and glycemic control in diabetes care, the synthesis data were grouped and presented thematically in the narrative review.

Critical Evaluation

The quality and relevance of the included studies were critically evaluated to verify the validity and trustworthiness of the material offered in the narrative review. Studies with methodological flaws or possible biases were considered in light of the larger body of knowledge.

Ethical Considerations

No human subjects, animal testing, or ethical issues were involved in this narrative review, which only relied on published literature.

Epidemiology of coexistence

Healthcare professionals, academics, and policymakers must comprehend the epidemiology of epilepsy and diabetes cohabitation. The prevalence of epilepsy in people with diabetes and vice versa is explored in this section, emphasizing age, gender, and demographic trends. It uses recent research to illuminate the intricate interactions between these two circumstances.

Prevalence of Epilepsy in Individuals with Diabetes

Diabetes and epilepsy are two common chronic health disorders with different pathophysiologies. Diabetes is defined by blood glucose levels that are out of control. The coexistence of multiple ailments has attracted increasing attention in recent years. Recent epidemiological studies have shown that people with diabetes have a higher risk of seizures than those without the condition. According to reports, people with diabetes have an epilepsy prevalence between 1.5 and 2 times higher than those without the disease [9,10]. Genetics, inflammation, and metabolic dysfunction are only a few common risk factors linked to this connection [11,12]. This comorbidity is significantly influenced by age. With advancing age, people with diabetes are more likely to acquire epilepsy [13]. In older populations, where both diabetes and epilepsy are more common, this age-dependent association is evident [14]. A combination of vascular and metabolic variables and age-related changes in brain structure and function causes this trend.

Diabetes Prevalence in People with Epilepsy

Contrarily, compared to the general population, those with epilepsy also have a higher prevalence of diabetes. Numerous research [15,16] have shown that people with epilepsy have a greater prevalence of diabetes, particularly type 2 diabetes. Numerous factors impact this comorbidity, including using specific AEDs, lifestyle choices, and shared genetic vulnerability [17,18].

Demographic Trends, Gender, and Age

Understanding the coexistence of epilepsy and diabetes depends on an individual's age. Age-related increases in epilepsy risk are observed, and this pattern is particularly pronounced in people with diabetes [19]. Epilepsy is more common in older persons with diabetes than younger adults, which may be connected to aging-related changes in brain structure and function. In this comorbidity's epidemiology, gender also has an impact. According to specific research, women with diabetes may be more likely than men to experience epilepsy [19]. However, gender-specific patterns are not constant across all studies, and more investigation is required to clarify this connection. Demographic factors like socioeconomic status and location may influence epilepsy and diabetes coexistence. According to studies [18,20], those from lower socioeconomic origins may be more likely to develop both illnesses.

Furthermore, the incidence of epilepsy and diabetes may fluctuate by geographical location due to variations in healthcare access, way of life, and genetic predisposition [21,22]. Recognizing the epidemiology of epilepsy and diabetes cohabitation has significant clinical consequences. Healthcare professionals must diligently assess and manage both illnesses in patients with this comorbidity, especially in older age groups. To guarantee the best results, comprehensive treatment strategies that cover both epilepsy and diabetes management are essential. Additionally, while organizing healthcare services and allocating resources, politicians and healthcare systems should consider the rising prevalence of these illnesses. The burden of this comorbidity may be lessened through screening programs and educational campaigns aimed at individuals with a higher risk of having epilepsy and diabetes.

In conclusion, complicated interactions impacted by age, gender, and demographic characteristics are revealed by the epidemiology of cohabitation between epilepsy and diabetes. Epilepsy is more likely to occur in people with diabetes, and the reverse is also true. Age-related patterns, gender differences, and socioeconomic considerations further shape the epidemiological landscape of this comorbidity. For clinical management and public health measures to be effective, it is essential to comprehend these patterns.

Mechanisms of interaction

Understanding the complicated interactions between these two medical diseases, including the bidirectional connections between epilepsy and diabetic management, is essential. This section explores the complex network of interactions, offering light on how seizures may affect glucose metabolism and glycemic management, as well as outlining possible interactions between diabetes and epilepsy, including increased brain excitability and oxidative stress.

Effect of Seizures on Glucose Metabolism

Epilepsy's signature symptom, seizures, is a dynamic physiological phenomenon significantly impacting many internal processes, including glucose metabolism. Several mechanisms are involved in the complex link between seizures and glycemic management. As part of the body's reaction to the epileptic event, seizures cause the production of stress hormones such as cortisol and adrenaline [23]. These hormones aid in developing insulin resistance, which impairs the peripheral tissues' ability to absorb glucose [24]. Epileptic seizures may interfere with the autonomic nervous system, causing changes in the heart rate, blood pressure, and other physiological parameters [20]. Because of this imbalance, insulin secretion and sensitivity may be impacted, further compromising glycemic control [23]. Neurotransmitter levels in the brain can change due to epileptic seizures, which might impact nutritional preferences and hunger control [21]. This can worsen glycemic instability by causing changes in food intake and macronutrient composition. AEDs such as valproate and carbamazepine affect insulin sensitivity and glucose metabolism [25]. The glycemic control of people with epilepsy who take these drugs may be disturbed. Clinicians who treat individuals with diabetes and epilepsy must comprehend these pathways. The possibility of acute glucose dysregulation during and after seizures should be considered in proper therapy.

Diabetes's Effects on Epilepsy: Oxidative Stress and Altered Brain Excitability

On the other hand, diabetes can significantly impact how epilepsy develops and is treated. Changes in brain excitability and oxidative stress are two essential ways diabetes affects epilepsy. Increased neuronal excitability and synaptic transmission are characteristics of altered brain excitability related to diabetes [25]. It has been demonstrated that hyperglycemia, a defining feature of diabetes, lowers seizure thresholds, rendering people more prone to seizures [26]. Due to this increased brain excitability, there may be a higher chance that seizures will start and repeat. Oxidative stress, or an imbalance between the body's antioxidant defense mechanisms and the formation of reactive oxygen species (ROS), is frequently associated with diabetes [27]. Oxidative stress can contribute to neuronal malfunction and damage, perhaps making epilepsy more severe.

Furthermore, oxidative stress can affect how well AEDs work [27]. It is significant to highlight that several variables, including the duration and severity of diabetes, glycemic management, and individual sensitivity, affect how diabetes affects epilepsy. As a result, people with diabetes, especially those with poor glycemic control, may be more likely to experience seizures and have trouble managing them. Additionally, the usage of antidiabetic drugs may have an impact on epilepsy. By modifying mitochondrial activity and oxidative stress, some drugs, including metformin, have shown potential neuroprotective effects in epilepsy [28].

On the other hand, sulfonylureas, another family of antidiabetic medications, has been linked to a higher risk of hypoglycemia, which can lower seizure thresholds [26]. In conclusion, complex systems that affect both epilepsy and glycemic control are involved in the bidirectional interactions between them. Cases can impair glucose metabolism and glycemic control through the production of stress hormones, disruption of the autonomic nervous system, changes in neurotransmitter levels, and the actions of some AEDs. On the other hand, diabetes can cause oxidative stress and increased brain excitability, thus raising the risk and severity of epilepsy. These interactions highlight the value of a multidisciplinary approach to managing people with epilepsy and diabetes, considering both illnesses in treatment plans and carefully monitoring glucose control and seizure activity.

Shared risk factors

Discovering the intricate connections between these two medical disorders requires understanding the risk factors contributing to diabetes and epilepsy. The common genetic, environmental, and lifestyle risk factors for epilepsy and diabetes will be examined in detail in this part, highlighting the complex interactions among these risk factors.

Genetic Factors

A person's genetic makeup primarily affects epilepsy and diabetes. An individual's risk can be significantly increased by having a family history of either ailment. Even though they frequently differ, several susceptibility genes have been discovered for both illnesses. Ion channels, neurotransmitter receptors, and synaptic protein genes have all been linked to epilepsy [29]. Contrarily, genetic variations affecting insulin secretion and resistance impact diabetes [29,30]. Some genes, such as KCNJ11 and ABCC8, have been linked to both diseases, albeit [30]. There is mounting evidence of similar genetic pathways that underlie epilepsy and diabetes and unique susceptibility genes. For instance, these diseases have been linked to inflammatory, oxidative stress, and insulin signaling pathways [31]. These converging pathways could be why people with one ailment may be more susceptible to developing the other.

Environmental Factors

Chronic inflammation, a frequent environmental component, facilitates epilepsy and diabetes [30,32]. Inflammatory processes can alter glucose metabolism, raise the likelihood of insulin resistance in people with diabetes, and promote brain hyperexcitability, which may cause seizures in people with epilepsy [32]. Epilepsy and diabetes are two conditions that can develop in children exposed to harmful environmental factors during pregnancy, such as maternal gestational diabetes or exposure to chemicals [32]. These early-life exposures can influence the developing brain, increase the risk of later-life seizures, and harm metabolic health.

Lifestyle Variables

Obesity is a recognized risk factor for both epilepsy and diabetes. Type 2 diabetes and insulin resistance are linked to excess body weight [26]. Additionally, metabolic changes brought on by obesity can affect how the brain functions, thus raising the risk of seizures [8]. Obesity, insulin resistance, and type 2 diabetes are all caused by physical inactivity and a sedentary lifestyle [27]. In addition, a lack of exercise may impact neural function and brain health, raising the risk of seizures [33]. Diet can have an impact on both diabetes and epilepsy risk factors. Diets heavy in fat and sugar have been linked to a higher risk of type 2 diabetes [32]. The ketogenic diet, for example, has been proven to decrease seizure frequency in people with epilepsy, demonstrating the reciprocal association between diet and seizure control [34]. Chronic stress is a risk factor that both diabetes and epilepsy share for developing and worsening [32,33]. Increased cortisol levels and other stress-related hormonal changes may affect neuronal excitability and glucose metabolism, leading to seizures [35].

Comorbid Psychiatric Conditions

Both epilepsy and diabetes frequently co-occur with psychological comorbidities such as depression and anxiety [36]. These circumstances may worsen both of these disorders' management and prognosis. In addition, the ongoing stress of depression and anxiety may impact the previously mentioned common risk factors. Antipsychotic drugs, for example, have metabolic side effects that may raise the risk of diabetes when taken to treat psychiatric comorbidities. In addition, several psychiatric drugs can lower the seizure threshold and raise the likelihood of seizures in people with epilepsy [36]. Finally, the risk factors associated with diabetes and epilepsy have typically offered a robust framework for comprehending the intricate interactions between these two illnesses. Genetic predisposition, environmental factors like inflammation and gestational exposures, lifestyle factors like obesity, sedentary behavior, food, stress, psychiatric comorbidities, and associated medications all influence vulnerability to these diseases. Understanding these common risk factors is essential for healthcare professionals since it guides holistic patient care, early intervention, and preventative initiatives.

Impact on diabetes management

An intricate clinical difficulty is managing diabetes in people with concomitant epilepsy. The particular difficulties that healthcare professionals have when managing diabetes for these people are covered in this section. We will also discuss the importance of antidiabetic drugs, their possible influence on seizure control, the acute effects of seizures on blood glucose levels, and the implications for therapy.

Diabetes Management Challenges for People with Epilepsy

Epileptic seizures may adversely impact blood sugar levels. The production of stress hormones like cortisol and adrenaline during a seizure might result in hyperglycemia [1,2]. On the other hand, postictal states could cause hypoglycemia because of increased glucose use during seizures. For people with epilepsy, it can be difficult to achieve stable glycemic control due to these glycemic oscillations. The efficacy and safety of antidiabetic treatments may be impacted by interactions with AEDs, frequently used to treat seizures. For instance, several AEDs, including carbamazepine and phenytoin, might cause hepatic enzyme induction, which may lessen the efficiency of oral hypoglycemic medications [6].

On the other hand, several antidiabetic drugs may change AED concentrations, resulting in less-than-ideal seizure control [7,8]. Epilepsy-related cognitive deficits and behavioral problems can make managing diabetes challenging. Executive function impairment, memory impairment, and medication adherence may make diabetes self-care activities like monitoring blood glucose levels, adhering to dietary recommendations, and following prescription regimens more challenging [15,20]. Psychiatric comorbidities like depression and anxiety are frequently more prevalent in people with concomitant epilepsy and diabetes [33]. These problems can impact mood, motivation, and self-care practices, making managing diabetes more difficult.

Seizures' Immediate Effects on Blood Glucose Levels

Seizures' immediate and long-term effects impact the management of diabetes and blood glucose levels. Stress chemicals like cortisol and adrenaline spike after a seizure. These hormones inhibit glucose absorption by peripheral tissues while triggering the release of glucose from the liver (gluconeogenesis). Blood glucose levels increase as a result [2,3]. Following a seizure, glucose usage may remain high, resulting in hypoglycemia (low blood sugar) [7]. It can be challenging to control this postictal state if the person is ignorant of the occurrence. When someone with epilepsy experiences these abrupt glycemic swings, their antidiabetic medication dosage must be carefully monitored and adjusted. Continuous glucose monitoring (CGM) systems can help identify these quick changes and direct the right actions.

Antidiabetic Drugs: Their Function and Effect on Seizure Control 

When choosing an antidiabetic drug for a person with epilepsy, it is essential to consider its ability to affect seizure management and any potential interactions with AEDs. The cornerstone of managing diabetes is still insulin. Insulin provides precise blood glucose control for people with epilepsy and is unaffected by interactions with AEDs [37]. But watching for hypoglycemia is essential, particularly before and after seizures. Individualized oral hypoglycemic drug selection should take both diabetes and epilepsy management into account. By modifying oxidative stress and mitochondrial function, some medications, such as metformin, have demonstrated potential neuroprotective effects in epilepsy [38]. Sulfonylureas, which might increase the risk of hypoglycemia, should only be used cautiously in people with epilepsy [38]. Glucagon-like peptide-1 receptor agonists (GLP-1 RAs) and sodium-glucose cotransporter 2 (SGLT2) inhibitors are two new antidiabetic drugs that show promise for improving glycemic control while posing less risk for hypoglycemia [37,38].

Further study is needed to determine whether they could positively or negatively affect seizures. Managing diabetes in people with epilepsy requires considering glycemic fluctuations during and after seizures, potential drug interactions between antidiabetic and antiepileptic medications, and the cognitive and behavioral difficulties frequently present in both conditions. To guarantee ideal glycemic control and reduce the risk of seizures, healthcare practitioners must use a tailored approach that incorporates the knowledge of epilepsy and diabetes specialists as needed. Comprehensive care for these patients must include ongoing observation, patient education, and psychosocial support.

Impact on epilepsy management

When treating patients with comorbid diseases, it is critical to comprehend how diabetes and glycemic control can affect the development and management of epilepsy. The delicate connection between diabetes, glycemic control, and epilepsy will be covered in this part, along with how hyperglycemia can start or worsen seizures. We will also review the factors that medical professionals must consider while selecting AEDs for diabetic patients.

Epilepsy and Diabetes Interaction

The hallmark of diabetes, hyperglycemia, has been linked to a reduced seizure threshold. Increased neuronal excitability in the brain can increase blood glucose levels, rendering people more susceptible to seizures [1]. In those with epilepsy, this increased excitability can lead to the beginning of new episodes or an increase in seizure frequency. Glucose serves as a significant source of energy for the brain. Diabetes-related glucose metabolism dysregulation has been linked to altered brain activity and increased neuronal excitability [10]. Fluctuations in blood sugar levels, especially when not properly managed, might cause seizures or exacerbate epilepsy that already exists. Oxidative stress and neuroinflammation are frequently linked to diabetes and harm brain health [14]. The pathophysiology of epilepsy has been linked to these mechanisms, demonstrating the interaction between the two diseases [36].

Having High Blood Sugar Can Cause Seizures

Increased blood glucose levels have been demonstrated to encourage the release of proinflammatory cytokines, which have been shown to lower seizure thresholds and increase neuronal excitability [33]. An imbalance of the excitatory neurotransmitter glutamate can result from hyperglycemia. Because of this imbalance, there may be excessive neuronal activation, which raises the risk of seizures [34]. Variations in glucose levels can affect how well ion channels function in neurons, which can upset the electrolyte balance. These alterations can cause neuronal hyperexcitability and seizures [35].

Factors to Take into Account When Choosing AEDs for Diabetics

Due to the possibility of interactions between AEDs and antidiabetic drugs, choosing the right AEDs for people with diabetes requires careful consideration. Some AEDs, such as carbamazepine and phenytoin, cause the hepatic enzymes that hasten the metabolism of antidiabetic medications like sulfonylureas to decrease their efficiency [39]. On the other hand, antidiabetic drugs' effects on their pharmacokinetics may impact AEDs' serum concentrations and therapeutic efficacy [39]. In people with diabetes, AEDs that lower the seizure threshold or encourage hypoglycemia should be avoided or taken cautiously. For instance, sulfonylureas should be prescribed cautiously to prevent potentially hazardous blood glucose swings since they can increase the risk of hypoglycemia [39]. By regulating oxidative stress and mitochondrial activity, some antidiabetic drugs, such as metformin, have shown potential neuroprotective effects [40]. These drugs may provide further advantages for those with concomitant epilepsy by perhaps lessening the intensity or frequency of seizures. Individualized epilepsy management is necessary for diabetic patients. When deciding on a course of treatment, medical professionals should take the patient's particular epilepsy syndrome, frequency of seizures, and reaction to AEDs into account. It is frequently required for diabetes and epilepsy professionals to work closely together and monitor both disorders regularly.

In conclusion, there are many different ways that diabetes, glycemic management, and epilepsy interact with one another. The significance of having the best possible diabetes control in people with epilepsy is highlighted because hyperglycemia can lower seizure thresholds and function as a potential seizure trigger. AEDs should be carefully chosen, considering any potential interactions with diabetes medicines and the danger of hypoglycemia. The most remarkable outcomes for patients with these comorbid illnesses require a specialized, multidisciplinary therapy approach that includes epilepsy and diabetes specialists.

Clinical management strategies

A thorough and multidisciplinary approach is necessary to manage the particular complications and interactions between concomitant epilepsy and diabetes. The importance of interdisciplinary collaboration between neurologists and endocrinologists, individualized treatment strategies, lifestyle changes, and the function of continuous glucose monitoring will all be covered in this section.

Ideal Clinical Strategies

Each patient with comorbid epilepsy and diabetes presents a unique clinical profile that includes the type and severity of epilepsy, diabetes management, and lifestyle factors. Care should, therefore, be highly customized. To comprehend each patient's unique demands and difficulties, healthcare professionals should undertake a thorough examination. Working together is essential for effective care between endocrinologists and neurologists. A multidisciplinary team can optimize drug schedules, keep an eye out for potential interactions, and make sure that treatment plans take both epilepsy and diabetes into account [24]. For coordinated treatment, regular contact between professionals is essential. Epilepsy and diabetes must be regularly monitored in people with comorbid illnesses. This entails regular neurological examinations, such as the frequency and severity of seizures, and ongoing glucose monitoring to keep track of glycemic changes. Healthcare professionals can modify treatment strategies in an educated manner through monitoring. For several reasons, interdisciplinary cooperation between neurologists and endocrinologists is crucial. Interactions between AEDs and antidiabetic treatments may impact drug efficacy and safety. To select AEDs and diabetes medications with the fewest interactions and side effects, neurologists and endocrinologists must collaborate [22]. Poor glycemic control can lower the seizure threshold, leading to seizures. Endocrinologists can assist in controlling blood glucose levels, lowering the possibility of complications from epilepsy.

Additionally, improved seizure management can improve the patient's general quality of life [26]. Collaborative care makes comprehensive lifestyle treatments possible. Healthcare professionals can discuss food, exercise, and weight management with patients who have both diseases. Lifestyle changes are essential for glycemic control and can affect the frequency of seizures [28].

Customized Treatment Programs

The possibility of drug interactions and adverse effects should be considered when choosing antiepileptic and antidiabetic drugs. For instance, avoiding AEDs with documented hypoglycemic effects can assist people with diabetes in avoiding hazardous blood glucose reductions [4]. It is crucial to recognize and treat seizure causes, such as hyperglycemia. Regularly checking blood sugar levels and taking antidiabetic drugs as prescribed will help stabilize glycemic levels and lessen the risk of seizures caused by hyperglycemia [38]. An individual's mental health may suffer as a result of having both diabetes and comorbid epilepsy. People with access to psychosocial support, such as counseling and support groups, can better manage the difficulties of managing both diseases [33].

Lifestyle changes: Maintaining a healthy weight is essential for managing diabetes and epilepsy. Healthcare professionals and nutritionists can work together to create individualized nutrition regimens that encourage weight control and consider the particular dietary requirements of people with diabetes and epilepsy [34]. Regular exercise can increase glycemic control and insulin sensitivity. To reduce their risk of seizures, people with epilepsy should follow safe exercise procedures. Planning is critical and should consider the timing and type of exercise [32]. Ongoing stress can exacerbate both epilepsy and diabetes. To assist people in coping with stress better, the treatment plan can include stress management strategies such as mindfulness meditation and relaxation activities [40].

Continuous glucose monitoring: CGM offers real-time information on blood glucose levels, assisting patients and medical professionals in spotting trends and promptly modifying prescription schedules and lifestyle choices [40]. CGM can detect impending hypoglycemia, allowing early intervention to stop dangerous blood sugar dips. This is crucial for people who are susceptible to hypoglycemia-related seizures. According to studies, CGM can help people with diabetes achieve better glucose control and lower their risk of hypoglycemia episodes [38]. In conclusion, treating diabetes with concomitant epilepsy requires a multidisciplinary approach. To address pharmaceutical interactions, enhance treatment regimens, and efficiently monitor both disorders, interdisciplinary collaboration between neurologists and endocrinologists is necessary. Individuals dealing with the difficulties of these coexisting disorders can significantly enhance their quality of life with the help of individualized treatment plans, lifestyle changes, and continuous glucose monitoring.

Future directions and research gaps

Even though substantial progress has been made in understanding the connection between epilepsy and glycemic management, several knowledge gaps still require further investigation. This section will highlight the research gaps and prospective directions for further study and clinical trials.

Epilepsy-Diabetes Connection: Mechanisms

Epilepsy and diabetes are recognized to interact, but the precise underlying mechanisms are still poorly understood. Clarifying the molecular and cellular processes that link these two diseases will require more study. How can seizures affect the metabolism of glucose, and how does diabetes affect the excitability and susceptibility to seizures of neurons? The complex mechanisms underlying how epilepsy and diabetes interact can be uncovered through experimental research employing animal models. Researchers can examine how neurotransmitters, stress hormones, and inflammatory markers mediate the link. Additionally, modern neuroimaging methods like functional MRI and positron emission tomography can be used to examine how the brain uses glucose and the activity of its neurons in people with both disorders.

Specific Therapies

Creating specialized medicines that cater to the particular requirements of people with comorbid epilepsy and diabetes is required. There is little research on integrated therapeutic techniques, and current treatment modalities frequently concentrate on treating each issue separately. Clinical trials should be created to assess the efficacy of combined therapeutic modalities that simultaneously treat diabetes and epilepsy. The use of antidiabetic drugs with possible neuroprotective properties (such as metformin) and their influence on seizure management should be considered in these investigations. Additionally, research into cutting-edge therapeutics that address typical underlying mechanisms, like oxidative stress and inflammation, may result in ground-breaking cures.

Personalized Treatment Programs

An emerging field of research is the creation of individualized treatment programs for people with both illnesses. Care must be tailored to each patient's clinical profile, including epilepsy, glycemic management, and lifestyle factors. The development of recommendations and algorithms for the customized management of diabetes and concomitant epilepsy should be the main topic of future study. These recommendations ought to take the patient's age, gender, genetic propensity, and psychological factors into account. Research should also examine how telemedicine and digital health tools might help underserved or rural populations receive individualized care.

Impact on Cognition and Psychosocial

Further research is needed to determine how epilepsy and diabetes interact cognitively and socially. Both illnesses have the potential to cause cognitive decline, sadness, and anxiety, all of which harm a person's quality of life. To evaluate the cognitive and psychosocial outcomes of people with comorbid epilepsy and diabetes over time, longitudinal studies are required. In this research, the effects of glycemic management on mental health and cognitive performance should be assessed. Interventions for preventing cognitive decline and enhancing general well-being should also be identified.

Diabetic Epilepsy Prevention

There is little research on preventive measures, even though managing epilepsy in people with diabetes has received much attention. An important area of research is identifying risk factors and treatments that can lower the prevalence of epilepsy in people with diabetes. In diabetes patients, modifiable risk factors that increase their vulnerability to epilepsy should be sought after in epidemiological investigations. Glycemic control, lifestyle elements (such as nutrition and exercise), and elements of the metabolic syndrome may all be risk factors. The effectiveness of therapies like lifestyle changes and neuroprotective drugs in lowering the risk of epilepsy in this cohort can be assessed through clinical studies.

Models for Delivering Healthcare

Innovative healthcare delivery strategies that enable interdisciplinary collaboration between neurologists and endocrinologists must be investigated. Optimizing outcomes for people with comorbid diseases requires integrated care models that simplify communication and coordination. The success of various healthcare delivery modalities, such as telemedicine and collaborative care clinics, should be the main subject of research. The results of this research should be used to generate best practices for treating patients with comorbid epilepsy and diabetes by evaluating patient satisfaction, clinical outcomes, and cost-effectiveness.

In conclusion, the interaction between epilepsy and glycemic control is a complicated and developing area that presents many study prospects. Addressing the research gaps above can result in better comprehension, more efficient therapies, and better results for those with comorbid epilepsy and diabetes. Collaboration across researchers, doctors, and healthcare systems is crucial to expanding our understanding and improving the care of these people.

## Conclusions

This narrative review explores the complex interaction between epilepsy and diabetes, highlighting the clinical importance of this link. Bidirectional impacts are one of the key insights: epilepsy can lower seizure thresholds due to neuronal excitability caused by hyperglycemia, while diabetes can affect brain function, complicating the therapy of epilepsy. Comorbidity is a result of common risk factors like heredity and lifestyle. Comorbid cases are challenging to manage since hyperglycemia can cause seizures, necessitating careful medication choice. Neurologists and endocrinologists need to work together interdisciplinary. Holistic care depends heavily on lifestyle changes like stress reduction and weight management. Real-time changes are aided by continuous glucose monitoring. Therapy plans that consider each patient's unique characteristics, epilepsy type, and glycemic control are crucial. There are still unanswered questions regarding mechanisms, specific treatments, and prevention measures. Clinical practice must recognize and treat this complex interaction, necessitating an interdisciplinary strategy, ongoing supervision, and specialized care. The management of comorbidities must be improved by ongoing study.
